# Is transanal irrigation the best treatment possibility for low anterior resection syndrome? A multicenter, randomized clinical trial: study protocol

**DOI:** 10.3389/fsurg.2024.1384815

**Published:** 2024-05-13

**Authors:** Michail Klimovskij, Ignas Civilka, Andrej Aleinikov, Tomas Aukstikalnis, Peter Christensen, Audrius Dulskas

**Affiliations:** ^1^ Department of Colorectal Surgery, East Sussex Healthcare Trust, Hastings, United Kingdom; ^2^ Faculty of Medicine, Vilnius University, Vilnius, Lithuania; ^3^ Department of Surgical Oncology, National Cancer Institute, Vilnius, Lithuania; ^4^ Department of Rehabilitation Physical and Sports Medicine, Faculty of Medicine, Institute of Health Sciences, Vilnius University, Vilnius, Lithuania; ^5^ Department of Surgery, Aarhus University Hospital, Aarhus, Denmark; ^6^ Danish Cancer Society Centre for Research on Survivorship and Late Adverse Effects After Cancer in the Pelvic Organs, Aarhus University Hospital, Aarhus, Denmark

**Keywords:** fecal incontinence, treatment of bowel dysfunction, colorectal surgery, low anterior resection syndrome, quality of life, transanal irrigation

## Abstract

**Background:**

Up to 50% of patients who undergo rectal resection suffer from various and partly severe functional problems, despite the preservation of the anal sphincter. These complaints are defined as low anterior resection syndrome (LARS). So far, there are no randomized clinical trials regarding the most effective treatment for LARS. Our aim is to evaluate whether transanal irrigation improves bowel function and quality of life in patients following low anterior resection compared to best supportive care.

**Methods:**

Patients who have undergone low anterior resection will be approached for this study. On patient's visit, complaints regarding the defecation as well as any deterioration in their overall quality of life will be assessed using questionnaires such as the Low Anterior Resection Syndromes score, Wexner score, European Organization for Research and Treatment of Cancer (EORTC) Quality of Life (QOL) CR-29, and Measure Yourself Medical Outcome Profile tool. Few additional target questions will be also asked, such as “Would you recommend the treatment to anybody; did you expect the improvement following the treatment; etc.” Questionnaires and scales will be filled on follow-up visits every 3 months for 1 year.

**Discussion:**

This multicenter, randomized controlled trial will lead to a better understanding of LARS treatment. Moreover, it will be a hypothesis-generating study and will inform areas needing future prospective studies.

**Clinical Trial Registration:**

ClinicalTrials.gov, identifier (NCT05920681).

## Introduction

Neoadjuvant treatment with low anterior resection and the formation of anastomosis provides excellent oncological results and is currently the gold standard of rectal cancer treatment (1). Preoperative chemoradiotherapy, vascular dissection, and surgical removal of the rectum and mesorectum cause significant colorectal motility impairment. This results in a variety of symptoms (multiple bowel movements, recurrent urge episodes, hoarding, urinary, fecal incontinence, etc.) that are associated with severe impairment of quality of life. These complaints are summarized as low anterior resection syndrome (LARS) (2). Moreover, surgery may lead to increased morbidity, prolonged hospital stay, readmission, sepsis, and death (3, 4).

Our previous studies showed the prevalence of LARS following rectal surgery in Lithuania reaches up to 75% (5). More importantly, it is a long-term effect—50% of patients have these symptoms 5 or more years after the surgery (6).

Transanal irrigation (enema) (TAI) is a promising treatment modality for LARS patients with increasing prospective data from published studies (7). Only a few clinical trials have been published in the literature where the benefit of transanal irrigations in the treatment of LARS has been investigated (8–10). In 2018, a study conducted in Italy (8), with 27 patients, evaluated transanal irrigation as a potentially beneficial treatment modality in the treatment of LARS. It was shown that the use of TAI demonstrates notable efficacy in treating LARS and resulted in improved continence and quality of life. However, this study included patients with chronic LARS as well as patients with early symptoms following the surgery. Some patients’ symptoms may improve over the time following the operation, while others can suffer from colonic dysfunction and nerve damage, which are strongly related to the main symptoms of LARS, but do not seem to be influenced by time (8). Similar studies were conducted in Germany (2018 and 2023) (9, 10), which have also shown the benefit of transanal irrigation in controlling LARS, but due to time constraints, some patients refused this method. While patients treated by TAI showed significant improvements in bowel movements, a notable portion decided to stop the treatment and relied on supportive therapy only (9).

Despite these data, the use of TAI remains a matter of debate. In addition, there are only few randomized clinical trials so far comparing TAI with best supportive care (8, 9, 11, 12). Main limitations of this study are that a significant amount of patients (six patients, reduction of 27.27%) dropped out of the intervention (TAI) group (9), the small numbers, and the short follow-up period (8, 11, 12).

Thus far, there are no randomized clinical trials confirming or denying the hypothesis regarding the most effective treatment for LARS. Treatment recommendations for LARS have been published in 2021 (13). Here, authors propose to initiate the treatment with best supportive treatment. If it fails, transanal irrigations are started. Moreover, if this fails, invasive procedures are recommended (such as sacral nerve modulation or stoma). Some authors recommend the perineal stoma as an alternative stoma formation site in patients were sphincter preservation is not possible (14). However, data on the risk of LARS in this subgroup of patients is still lacking.

## Objectives

The primary objective of this study was to evaluate if transanal irrigation improves bowel function and quality of life in patients following low anterior resection compared with best supportive care.

Specific objectives were as follows:

1. To assess the proportion of patients with transanal irrigation that reduces the symptoms of LARS (decrease in absolute score).
2. To assess the proportion of patients with best supportive care that reduces the symptoms of LARS (decrease in absolute score).
3. Compare results between groups.

## Methods and analysis

### Study design

This is a multicenter randomized clinical trial. Centers from Lithuania (four centers), UK (at least one center), Denmark (at least one center), and other countries will be invited to participate through the European Society of Coloproctology’s (ESCP) trial map.

The main objective of this clinical trial is to evaluate whether transanal irrigation improves bowel function and quality of life in patients following low anterior resection best supportive care. This will be accomplished by recording the patient's complaints (defecation, urination problems, deterioration of quality of life) after the operation, filling the LARS score (3, 15), Wexner score (16), and quality of life questionnaires [European Organization for Research and Treatment of Cancer (EORTC) CR29 (17) and Measure Yourself Medical Outcomes Profile (MYMOP)] (18) with additional questions: Would you advice this treatment to anybody else? Did your quality of live improve? Did the bowel function improve? Are you satisfied with the treatment? Did you expect the treatment would help? These questions will be rated from 0 to 5. All will be filled in again during the visit every 3 months for 1 year.

### Study population

All patients who developed LARS and met the inclusion criteria will be offered participation in this clinical trial.

### Eligibility criteria

Inclusion criteria are as follows:

• Subject is an adult (≥18 years).
• Agrees to participate in a study.
• A low anterior resection (robotic, laparoscopic, or open) was performed [anastomosis up to 5–7 cm from the anocutaneous line when assessed with a finger or endo(recto)scope] following long-course (chemo)radiotherapy [the patients treated with 5-fluorouracil (FU) or capecitabine and radiation for 5 weeks; radiation therapy is given once a day at 1.8 Gy/day for a total of 50.0 Gy (1.8 Gy/fraction to the gross tumor and 45 Gy to pelvic lymph nodes)].
• >3 months have passed since the operation or the closure of the ileostomy (if formed).
• No anastomotic leak or stenosis (assessed clinically, during examination, and/or via a proctogram).
• LARS >30 points (major LARS).

Exclusion criteria are as follows:

• Tumor recurrence/progression.
• Pregnancy.
• Diagnosed with inflammatory bowel disease (ICD codes K50–59).
• Side-to-end anastomosis.
• Palliative care.
• Will not be able to perform irrigation.

### Recruitment

Patients with LARS and meeting the inclusion criteria will be offered participation in this clinical trial upon consultation with an abdominal surgeon or surgeon at the National Cancer Institute clinic (or any other participating center). The patient will be given time to think as much as necessary. All questions related to the clinical trial will be answered. The patient's decision to participate in the study or not will not have any effect on their further treatment and/or surveillance. Patients who have consented and signed the personal information form and the consent form will be included in the study. Only centers with a high volume of documented colorectal surgery can be included (centers performing at least 50 low anterior resections following neoadjuvant chemoradiotherapy per year may be assessed as candidates for participation).

### Informed consent

After patients are invited to participate in the study, they will be provided with information about this study. The research doctor or the person conducting the research authorized by the researcher provides information about the study to the subject. The doctor-researcher or the person conducting the research authorized by the researcher explains the information related to the research and the objectives of the research. If patients agree to participate in the study, they sign the informed consent form, indicating their name, surname, date, and time. The investigator will sign and date the consent form. The informed consent form is signed in two copies, one is given to the patient participating in the study and the other is kept at the study center (National Cancer Institute), in a secure place, with limited access to only the personnel of this biomedical study. The principal investigator is responsible for the storage of research documents.

### Randomization

Individuals participating in this study will be randomly divided into groups A and B by randomization (computer-generated random numbers and sealed envelopes will be used). The probability of falling into one or the other group is equal.

• Group A. This is a group of patients who will be subjected to transanal irrigation (experimental).
• Group B. This is a group of patients who will receive only the best currently in use maintenance treatment (control).

During the research, the name and surname of the subject will be replaced by a special code, restricting the identity of the subject. This code will be used in all study documents except the consent form. Only the main researcher and his representatives will have access to these data, and the staff performing the statistical analysis will not know which group the subject belongs to. Only the data analysts will be blinded to the patients groups (will get only data with names of Groups A and B, without any notification, of which group is which).

### Interventions

During the study, transanal irrigations will be used, which are considered safe procedures that do not pose additional risks to the patient.

#### Transanal irrigation

Transanal irrigation will be applied to patients who will enter the experimental group. The patient lies on the left or right side depending on the main hand with the knees bent. With the main hand, the TAI tip (cone catheter) lubricated with a lubricant is carefully introduced. The TAI bag (Coloplast Peristeen Transanal Irrigation System) is filled with warm water—it can be boiled or just from the tap. The contents of the TAI bag are slowly administered through the anus—the cone catheter is inserted [the starting volume is 500 ml of warm (around body temperature) water and it can be increased up to 1 L eventually over a 3- to 4-week period]. The duration of the TAI is about 15–20 min. Afterward, the subject goes to defecate until the bowel is empty. This action should be repeated daily.

The patients will be instructed by the treating physician and will be contacted within 3–4 weeks on the course of the procedure.

In case of bleeding or abdominal pain, patients were instructed to contact the team member at any time. For all other questions regarding TAI, the instructor could be contacted during office hours. All the adverse events and complications will be assessed weekly. In case of the high number of complications, the study will be ended.

#### Best supportive care

The control group will receive best supportive care: diet modification (low-fiber diet and personal recommendations were given), medications (bulk-forming agents and loperamide), and, if needed, diapers. All patients were instructed regarding the pelvic floor muscle training (Kegel exercise).

No patients received biofeedback therapy or any other interventions such as sacral nerve stimulation or percutaneous tibial nerve stimulation.

All the team members will be provided with the video teaching material and instructions for the procedures to be performed for both groups.

### Assessments

Data collection will take place during the patient visit. Demographic and clinical examination data will be collected from the medical documentation at the research center. During the visit, the patient's complaints after the operation (defecation, deterioration of quality of life) will be recorded, and LARS, Wexner scale, and quality of life questionnaires will be filled during the visit. Questionnaires and scales will be filled again during the visit every 3 months for 1 year. Other tests that will be performed during the visit will be long-term follow-up tests, an integral part of the treatment, not related to the clinical trial.

At the second stage, a longer follow-up will be planned—for more than 2 years after the end of treatment.

### Sample size

A sample size of 40 is planned (an improvement of 5 points on the LARS scale):

• 20 transanal irrigation group (experimental) and
• 20 best supportive care group (control).

To demonstrate a 5-point difference (with 80% certainty) between the intervention group and the control group, 34 patients were required to participate in the study (17 in each study arm). Taking into account a drop off of 20%, at least 20 patients per group will be needed. The primary endpoint was LARS score analyzed by unpaired *t* test.

### Outcome measures

The objectives of the trial are to evaluate in what proportion of patients (percentage) transanal irrigation and in what proportion of patients (percentage) the best supportive treatment reduces the symptoms of LARS score (change in absolute score), compare the results, and evaluate the statistical reliability. The secondary outcomes would be assessing the change in single LARS score's items.

### Data analysis

Statistical methods will be used for data analysis using the SPSS program.

### Statistical analysis

For statistical analysis, we will use the intention to treat principle.

## Data collection and management

### Data collection

Data obtained from demographic and clinical examination during the initial assessment and from the medical documentation at the study center will be collected. List of data to be obtained are subject's gender, age, height, weight, concomitant diseases, and medications used (their names and doses). The patient's complaints (defecation, urination problems, deterioration of quality of life) after surgery are recorded; LARS is evaluated according to questionnaires at each visit. The data collected via paper will later be uploaded into Excel, where it will be depersonalized. All questionnaires used have been validated (Wexner, LARS), or permission to use them has been obtained from the authors.

### Data management

All information will be recorded in electronic and paper documents created specifically for the clinical trial. No patient identifiable data (name, date of birth, address, etc.) will be recorded. Registered local investigators will have individual password-protected access to their unit's data entered into an electronic database. During the running of the audit, only local data will be visible to investigators; other sites’ data will not be accessible.

The main researcher, the research investigators, persons authorized by the ethics committees, or persons authorized by other controlling institutions will be able to get acquainted with the data collected for the purpose of the study, which allow the direct identification of the subject.

The data will be processed in a computerized manner, via electronic research documents and password-protected data (Appendix 1). Only the researchers know the access codes.

### Management and safety

During the study, transanal irrigation (enema) will be used—a safe procedure that may cause only minor inconveniences: longer delay in the morning toilet, nausea, abdominal bloating, increased bowel movements after the procedure, anus pain, or other rare side effects [e.g., intestinal perforation, which according to the literature occurs in 1 in 166,000 procedures (19, 20)].

These inconveniences will be recorded in the electronic journal. Moreover, some patients will receive the best supportive treatment, which is a safe treatment because it is non-interventional and therefore does not pose any additional risk to the patient.

## Discussion

In this study, we aim to evaluate if transanal irrigation improves bowel function and quality of life in patients following low anterior resection compared with best supportive care.

We have designed this study for a couple of reasons. Just recently, public guidelines were issued (13). The authors recommend conservative management as a first step. Even though there is little evidence that dietary modifications are effective for LARS patients, good results with a reduction of non-soluble fiber intake seems reasonable. The use of anti-diarrheal agents such as loperamide, if necessary, can also apply to LARS. The authors also recommend patient consultation before any treatment initiation and risk of LARS assessment. Moreover, all the dietary instructions or medications were prescribed just after the surgery. Together with best supportive treatment, pelvic floor muscle training with biofeedback may be advised. If “conservative” treatments are not helpful, patients may be advised to use TAI, sacral neuromodulation (SNM), or percutaneous tibial nerve stimulation. TAI seems to be a promising treatment modality. It has at least two benefits—first, as the bowel following irrigation is empty, the patient will have pseudocontinence; second, the bowel is “taught” to do the defecation movements at same time. Only a few randomized clinical trials are present comparing TAI vs. best supportive treatment (8–13). Main limitations of these trials are the small sample size and relatively short follow-up.

Therefore, our study will be the first international multicenter study including patients with LARS and using transanal irrigation. Moreover, a patient representative was included in the protocol recommending to choose the correct questions and questionnaires.

## Trial status

The first patient was included in August 2023. At the time of protocol revision (January 2024), two centers in Lithuania, one in Portugal, and one in UK are actively recruiting patients for the study, and 24 patients have already been included.

## Data Availability

All the data will be accessible from the corresponding author upon reasonable request.

## Appendix 1

SPIRIT 2013 Checklist: Recommended items to address in a clinical trial protocol and related documents^a^.

**Table:**

Section/item	Item no.	Description	Addressed on page number
Administrative information			
Title	1	Descriptive title identifying the study design, population, interventions, and, if applicable, trial acronym	_____1_____
Trial registration	2a	Trial identifier and registry name. If not yet registered, name of intended registry	1
	2b	All items from the World Health Organization Trial Registration Data Set	__________
Protocol version	3	Date and version identifier	_____1_____
Funding	4	Sources and types of financial, material, and other support	_____8_____
Roles and responsibilities	5a	Names, affiliations, and roles of protocol contributors	_____1_____
	5b	Name and contact information for the trial sponsor	_____1_____
	5c	Role of study sponsor and funders, if any, in study design; collection, management, analysis, and interpretation of data; writing of the report; and the decision to submit the report for publication, including whether they will have ultimate authority over any of these activities	None __________
	5d	Composition, roles, and responsibilities of the coordinating centre, steering committee, endpoint adjudication committee, data management team, and other individuals or groups overseeing the trial, if applicable (see Item 21a for data monitoring committee)	_____8_____
Introduction			
Background and rationale	6a	Description of research question and justification for undertaking the trial, including summary of relevant studies (published and unpublished) examining benefits and harms for each intervention	_____3_____
	6b	Explanation for choice of comparators	_____4_____
Objectives	7	Specific objectives or hypotheses	_____4_____
Trial design	8	Description of trial design including type of trial (e.g., parallel group, crossover, factorial, single group), allocation ratio, and framework (e.g., superiority, equivalence, noninferiority, exploratory)	_____5–7_____
Methods: participants, interventions, and outcomes			
Study setting	9	Description of study settings (e.g., community clinic, academic hospital) and list of countries where data will be collected. Reference to where list of study sites can be obtained	_____4_____
Eligibility criteria	10	Inclusion and exclusion criteria for participants. If applicable, eligibility criteria for study centres and individuals who will perform the interventions (e.g., surgeons, psychotherapists)	_____4_____
Interventions	11a	Interventions for each group with sufficient detail to allow replication, including how and when they will be administered	_____4_____
	11b	Criteria for discontinuing or modifying allocated interventions for a given trial participant (e.g., drug dose change in response to harms, participant request, or improving/worsening disease)	_____4, 5_____
	11c	Strategies to improve adherence to intervention protocols, and any procedures for monitoring adherence (e.g., drug tablet return, laboratory tests)	________4,5_____
	11d	Relevant concomitant care and interventions that are permitted or prohibited during the trial	_____6_____
Outcomes	12	Primary, secondary, and other outcomes, including the specific measurement variable (e.g., systolic blood pressure), analysis metric (e.g., change from baseline, final value, time to event), method of aggregation (e.g., median, proportion), and time point for each outcome. Explanation of the clinical relevance of chosen efficacy and harm outcomes is strongly recommended	_____7_____
Participant timeline	13	Time schedule of enrolment, interventions (including any run-ins and washouts), assessments, and visits for participants. A schematic diagram is highly recommended (see Figure)	_____7_____
Sample size	14	Estimated number of participants needed to achieve study objectives and how it was determined, including clinical and statistical assumptions supporting any sample size calculations	_____7_____
Recruitment	15	Strategies for achieving adequate participant enrolment to reach target sample size	_____7_____
Methods: assignment of interventions (for controlled trials)			
Allocation:			
Sequence generation	16a	Method of generating the allocation sequence (e.g., computer-generated random numbers), and list of any factors for stratification. To reduce predictability of a random sequence, details of any planned restriction (e.g., blocking) should be provided in a separate document that is unavailable to those who enrol participants or assign interventions	_____5, 6_____
Allocation concealment mechanism	16b	Mechanism of implementing the allocation sequence (e.g., central telephone; sequentially numbered, opaque, sealed envelopes), describing any steps to conceal the sequence until interventions are assigned	_____5_____
Implementation	16c	Who will generate the allocation sequence, who will enrol participants, and who will assign participants to interventions	_____5_____
Blinding (masking)	17a	Who will be blinded after assignment to interventions (e.g., trial participants, care providers, outcome assessors, data analysts), and how	_____5_____
	17b	If blinded, circumstances under which unblinding is permissible, and procedure for revealing a participant's allocated intervention during the trial	_____5_____
Methods: data collection, management, and analysis			
Data collection methods	18a	Plans for assessment and collection of outcome, baseline, and other trial data, including any related processes to promote data quality (e.g., duplicate measurements, training of assessors) and a description of study instruments (e.g., questionnaires, laboratory tests) along with their reliability and validity, if known. Reference to where data collection forms can be found, if not in the protocol	_____7_____
	18b	Plans to promote participant retention and complete follow-up, including list of any outcome data to be collected for participants who discontinue or deviate from intervention protocols	_____7_____
Data management	19	Plans for data entry, coding, security, and storage, including any related processes to promote data quality (e.g., double data entry; range checks for data values). Reference to where details of data management procedures can be found, if not in the protocol	_____7, 8_____
Statistical methods	20a	Statistical methods for analysing primary and secondary outcomes. Reference to where other details of the statistical analysis plan can be found, if not in the protocol	_____7, 8_____
	20b	Methods for any additional analyses (e.g., subgroup and adjusted analyses)	_____7, 8_____
	20c	Definition of analysis population relating to protocol non-adherence (e.g., as randomised analysis), and any statistical methods to handle missing data (e.g., multiple imputation)	_____7, 8_____
Methods: Monitoring			
Data monitoring	21a	Composition of data monitoring committee (DMC); summary of its role and reporting structure; statement of whether it is independent from the sponsor and competing interests; and reference to where further details about its charter can be found, if not in the protocol. Alternatively, an explanation of why a DMC is not needed	_____–_____
	21b	Description of any interim analyses and stopping guidelines, including who will have access to these interim results and make the final decision to terminate the trial	_____7_____
Harms	22	Plans for collecting, assessing, reporting, and managing solicited and spontaneously reported adverse events and other unintended effects of trial interventions or trial conduct	_____7_____
Auditing	23	Frequency and procedures for auditing trial conduct, if any, and whether the process will be independent from investigators and the sponsor	_____8_____
Ethics and dissemination			
Research ethics approval	24	Plans for seeking research ethics committee/institutional review board (REC/IRB) approval	_____8_____
Protocol amendments	25	Plans for communicating important protocol modifications (e.g., changes to eligibility criteria, outcomes, analyses) to relevant parties (e.g., investigators, REC/IRBs, trial participants, trial registries, journals, regulators)	_____–_____
Consent or assent	26a	Who will obtain informed consent or assent from potential trial participants or authorised surrogates, and how (see Item 32)	_____8_____
	26b	Additional consent provisions for collection and use of participant data and biological specimens in ancillary studies, if applicable	_____8_____
Confidentiality	27	How personal information about potential and enrolled participants will be collected, shared, and maintained in order to protect confidentiality before, during, and after the trial	_____8_____
Declaration of interests	28	Financial and other competing interests for principal investigators for the overall trial and each study site	_____8_____
Access to data	29	Statement of who will have access to the final trial dataset, and disclosure of contractual agreements that limit such access for investigators	_____8_____
Ancillary and post-trial care	30	Provisions, if any, for ancillary and post-trial care, and for compensation to those who suffer harm from trial participation	_____–_____
Dissemination policy	31a	Plans for investigators and sponsor to communicate trial results to participants, healthcare professionals, the public, and other relevant groups (e.g., via publication, reporting in results databases, or other data sharing arrangements), including any publication restrictions	_____–_____
	31b	Authorship eligibility guidelines and any intended use of professional writers	_____–_____
	31c	Plans, if any, for granting public access to the full protocol, participant-level dataset, and statistical code	_____–_____
Appendices			
Informed consent materials	32	Model consent form and other related documentation given to participants and authorised surrogates	___Appendix 1___
Biological specimens	33	Plans for collection, laboratory evaluation, and storage of biological specimens for genetic or molecular analysis in the current trial and for future use in ancillary studies, if applicable	___Appendix 2___

^a^ It is strongly recommended that this checklist be read in conjunction with the SPIRIT 2013 Explanation & Elaboration for important clarification on the items. Amendments to the protocol should be tracked and dated. The SPIRIT checklist is copyrighted by the SPIRIT Group under the Creative Commons “Attribution-NonCommercial-NoDerivs 3.0 Unported” license.

## Appendix 2

SUTIKIMAS DALYVAUTIBIOMEDICININIAME TYRIME

(pavyzdinė forma, palikti tik tyrimui aktualią informaciją, žr. išnašas)

1. Aš perskaičiau šią Informuoto asmens sutikimo formą ir supratau man pateiktą informaciją.
2. Man buvo suteikta galimybė užduoti klausimus ir gavau mane tenkinančius atsakymus.
3. Supratau, kad galiu bet kada pasitraukti iš tyrimo, nenurodydama(s) priežasčių.^1^
4. Supratau, kad asmuo, dėl kurio dalyvavimo biomedicininiame tyrime aš duodu sutikimą, gali bet kada pasitraukti iš tyrimo, nenurodydamas priežasčių.^2^
5. Supratau, kad norėdama(s) atšaukti sutikimą dalyvauti biomedicininiame tyrime, raštu turiu apie tai informuoti tyrėją/kitą jo įgaliotą biomedicininį tyrimą atliekantį asmenį.
6. Patvirtinu, kad turėjau užtektinai laiko apsvarstyti man suteiktą informaciją apie biomedicininį tyrimą.
7. Supratau, kad dalyvavimas šiame tyrime yra savanoriškas.
8. Patvirtinu, kad sutikimą dalyvauti šiame biomedicininiame tyrime duodu laisva valia.
9. Leidžiu naudoti asmens duomenis ta apimtimi ir būdu, kaip nurodyta Informuoto asmens sutikimo formoje.
10. Patvirtinu, kad gavau Informuoto asmens sutikimo formos egzempliorių, pasirašytą tyrėjo/ kito jo įgalioto biomedicininį tyrimą atliekančio asmens.

**Table:**

Asmuo (ar kitas sutikimą turintis teisę duoti asmuo)					
				* *	_:_
vardas	pavardė	atstovavimo pagrindas	parašas	pasirašymo data	pasirašymo laikas

Patvirtinu, kad suteikiau informaciją apie biomedicininį tyrimą aukščiau nurodytam asmeniui.

Patvirtinu, kad asmeniui (ar kitam sutikimą duoti turinčiam teisę asmeniui) buvo skirta pakankamai laiko apsispręsti dalyvauti biomedicininiame tyrime, atsižvelgiant į biomedicininio tyrimo pobūdį, taip pat įvertinus kitas aplinkybes, galinčias daryti įtaką priimamam sprendimui.

Aš skatinau asmenį (ar kitą sutikimą turintį teisę duoti asmenį) užduoti klausimus ir į juos atsakiau.

**Table:**

Tyrėjas ar kitas jo įgaliotą biomedicininį tyrimą atliekantis asmuo					
			* *		_:_
